# A public health approach to eating disorders prevention: It’s time for public health professionals to take a seat at the table

**DOI:** 10.1186/1471-2458-12-854

**Published:** 2012-10-09

**Authors:** S Bryn Austin

**Affiliations:** 1 Department of Society, Human Development and Health, Harvard School of Public Health, Boston, MA, USA; 2 Division of Adolescent and Young Adult Medicine, Boston Children’s Hospital, 300 Longwood Ave, Boston, 02115, MA, USA

**Keywords:** Public health, Eating disorders, Prevention, Environment

## Abstract

**Background:**

The societal burden of eating disorders is clear, and though there is a compelling need for a public health approach to eating disorders prevention, public health professionals have yet to take up the challenge.

**Discussion:**

The article lays out an argument for what steps need to be taken to bring a public health approach to eating disorders prevention. First, stock is taken of what the field has achieved so far, using tools from the prevention science literature, and, second, a research plan of action is offered that plays to the unique strengths of public health, drawing on a triggers-to-action framework from public health law. Minimal participation was found from public health professionals in eating disorders prevention research, and the vast majority of prevention research to date was found to be concentrated within the disciplines of psychology and psychiatry. Extreme disciplinary concentration of the research has led to a preponderance of individually targeted prevention strategies with little research focused on environmental targets, particularly at the macro level. New environmental initiatives are now emerging, such as a government-sponsored mass media anti-dieting campaign, and legal bans on extremely thin models in advertising, but for the most part, they have yet to be evaluated. A triggers-to-action framework, which focuses on evidentiary base, practical considerations, and political will, developed in public health law provides a basis for a strategic research plan for a public health approach to eating disorders prevention.

**Summary:**

There is enormous potential for growth in the scope and diversity of eating disorder prevention research strategies, particularly those targeting the macro environment. A public health approach will require a strategic plan for research that leverages the macro environment for prevention. The full engagement of public health professionals will bring to the field the much broader range of preventive strategies and perspectives needed to tackle the problem of eating disorders.

## Background

The public health burden of eating disorders, including bulimia nervosa, anorexia nervosa, binge eating disorders, and the much more prevalent subthreshold variants, is clear. New evidence has documented eating disorder occurring across the globe in both developed and developing economies, [1] increasing rates of eating disorder symptoms and behaviors in both girls and boys, [2,3] high mortality rates of eating disorders, [4] and high treatment costs [5]. For years, calls have been mounting for health professionals to lead a public health approach to eating disorders prevention, [6-9] but still today, there is little sign that public health professionals have taken a seat at the table, much less helped to come up with a viable strategy for a public health approach to prevention.

It is obvious from even a cursory review of the eating disorders prevention literature that public health professionals largely have been sitting it out. In an informal survey of five recent scientific reviews [10-14] of eating disorders preventive interventions (including 87 unique papers), for 77.0% (n=67) of the articles, the first authors were based in departments of psychology or psychiatry, whereas for only 5.8% (n=5) of the articles did the first authors list any affiliation with a school or department of public health. Unquestionably, important advances in eating disorders prevention have been made without much contribution from public health professionals [10,14,15]. For instance, programs have used universal, selected, and indicated prevention approaches, been designed for children and adults, and targeted improvement of self-esteem and body esteem, rejection of unrealistic thinness ideals, enhancement of media literacy skills, and improved nutrition and physical activity; many have achieved some success [10,14,15]. But the extreme disciplinary concentration of the research and minimal participation from public health [7] has led to a preponderance of individually targeted prevention strategies with little research focused on environmental targets, particularly at the macro level [6,15-17]. Importantly, in the last few years, new macro-environmentally targeted initiatives are beginning to emerge, such as a government-sponsored mass media anti-dieting campaign and legal bans on extremely thin models depicted in advertising [18,19], but apparently only one of these types of initiatives has been subject to a systematic evaluation and published in the scientific literature [17].

In contrast to the chief goals of the clinical disciplines, prevention is the sine qua non of public health. The full engagement of public health in eating disorders prevention research could bring to the field the much broader range of preventive strategies and perspectives that will be essential to tackling the challenge of eating disorders. The reasons behind the general absence of public health professionals are likely complex but may relate to entrenched myths about the problem, including the mistaken beliefs that eating disorders affect only a small subset of females, that a focus on eating disorders will distract from obesity prevention, and that the practices of the diet-products and related industries are tangential to public health [7,20]. In addition, public health professionals and eating disorders specialists have few opportunities for physical or virtual proximity; they are usually employed in different contexts, publish in and read different journals, attend different professional conferences [15], and exposure to eating disorders research is rare in public health graduate training programs [7].

So what will it take to bring a public health approach to eating disorders prevention? First, it will require a careful review of the status of eating disorders prevention research, and then, a plan of action that capitalizes on the unique strengths of public health.

## Discussion

To get a sense of the current status of the field, the Prevention Maturation Schema, a systematic framework developed by public health professionals, can be used to guide assessment of the progress of a field toward prevention research [21]. The schema is based on the belief that achieving the public health goals of improving population health and reducing human suffering requires the sequential development of research. The five sequential phases of a field’s maturation toward prevention research are:

Phase 1: Establish link between risk behavior and health outcomes

Phase 2: Develop methods for measuring risk behavior and symptoms

Phase 3: Identify determinants of risk behavior and symptoms

Phase 4: Evaluate preventive interventions

Phase 5: Evaluate dissemination of preventive interventions

The schema can be applied to a field’s scientific literature to answer two questions: First, is a field ready for preventive intervention research (i.e., is enough known about the problem to begin prevention efforts)? Second, is productivity in preventive intervention research (Phases 4 and 5) comparable to the earlier phases (Phases 1–3)? Young fields tend to concentrate in the earlier phases and more mature fields in the later phases [21].

Adapting this schema, our team selected two international peer-review journals that are exclusively focused on eating disorders: *International Journal of Eating Disorders* (*IJED*) and *Eating Disorders: The Journal of Treatment and Prevention* (*EDJTP*). The former was selected because it is the oldest journal (founded in 1981) dedicated to eating disorders research and has the highest impact factor (2.8) of the eating disorders specialty journals. The latter was selected because its commitment to prevention research is clearly conveyed in the journal title.

With two independent coders, we reviewed all research articles (n=981) in both journals from January 2005 through December 2010 and coded the articles according to phase of prevention maturation. The intraclass correlation coefficient (ICC) for agreement between the two coders was strong (ICC 0.7; 95% confidence interval for ICC: 0.6, 0.8; P<0.0001).

**Figure 1:**
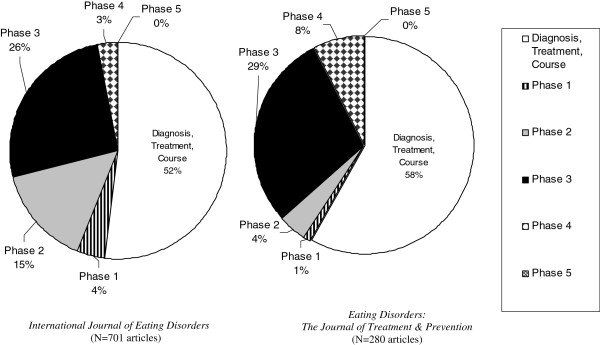
**Coding Results From Application of Prevention Maturation Schema to Articles Published in Two Eating Disorders Research Specialty Journals, 2005–2010 (N=981 Articles).** The Prevention Maturation Schema is structured in five sequential phases of research: Phase 1: Establish link between risk behavior and health outcomes; Phase 2: Develop methods for measuring risk behavior; Phase 3: Identify determinants of risk behavior; Phase 4: Evaluate preventive interventions; Phase 5: Evaluate dissemination of preventive interventions.

The findings were remarkably similar for the two journals (see Figure 1). First, more than half of the articles coded for each journal fell outside the schema altogether because they focused on topics such as psychiatric diagnostic classification of eating disorders, course after diagnosis, and treatment outcomes. Second, substantial development was observed through Phase 3 (identify determinants of risk behavior and symptoms), followed by a precipitous drop off in articles coded as Phase 4 (evaluate preventive interventions) or Phase 5 (evaluate dissemination of preventive interventions). For *IJED*, 3% of articles were coded as Phase 4 and none as Phase 5. For *EDJTP*, Phase 4 made up a greater percentage of articles (8%) than in *IJED*, but still no articles were coded as Phase 5. Returning to the two questions that the schema is designed to answer: Is the field of eating disorders ready for preventive intervention research? *Yes*: The substantial literature from Phase 1 through Phase 3 – and most especially the body of research on determinants of eating disordered behaviors and symptoms -- means the field is already well-prepared to concentrate on the next phase. Is productivity in Phases 4 and 5 comparable to Phases 1-3? *No*: In fact, the sharp drop after Phase 3 suggests that the field is ready for far more productivity in the prevention arena.

Admittedly, this application of the Prevention Maturation Schema is not exhaustive. These results are based on six years of articles from two journals, so eating disorders prevention research published in other years or other journals would not be included. In fact, enough Phase 4 eating disorders prevention research studies have been done – and often published in other journals – to support more than a few comprehensive reviews [10-14,22]. That said, apparently to date there has been only one large-scale dissemination research study (Phase 5) of an intervention shown to prevent eating disorder symptoms [23], and even if the proportion of articles in Phases 4 or 5 were double what we observed, the level of productivity in these phases would be substantially lower than that in Phase 3 or in the area of treatment and diagnosis.

The drop off in productivity at Phase 4 may in part relate to the complexity and expense of carrying out preventive interventions, particularly those with randomized controlled designs, and the challenges of obtaining funding. For instance, only a tiny fraction of the annual funding portfolio of the National Institute of Mental Health goes to primary prevention research while nearly 60% goes to brain research on causes of mental illness [24]. In addition, public health prevention scientists may be slow to recognize the relevance of eating disorders prevention to the mission of the field due to the reasons offered above [7,15].

To increase the field’s productivity in Phases 4 and 5 while bringing a public health approach to eating disorders prevention, we will need a strategic plan for research that plays to the strengths of public health. The field offers special expertise in tools for both leveraging forces in the macro environment for prevention and evaluating the effects of these strategies on prevention goals through systematic research. Many disciplines attend to facets of the micro and meso environments, some focusing on peer relationships, others emphasizing family, school settings, neighborhoods, while other disciplines focus on the macro environment, including the mass media, economy, law, and more [25-28]. Changes made in the macro environment have unparalleled potential for population-wide reach and high impact to improve population health, and the way we identify which changes achieve our prevention goals is through systematic evaluation research.

Examples of successful public health preventive interventions targeting the macro environment include changes in laws to require that seat belts be installed in cars by the manufacturers to reduce traffic fatalities, changes in tax law to increase sales tax on cigarettes to reduce teen smoking rates, improvements in agricultural sanitation requirements to reduce food-borne infections, and social marketing campaigns to change social norms to reduce drunk driving [28]. The tobacco, alcohol, fast food, and soda industries have been prime macro-environmental targets for public health initiatives [29-32]. The eating disorders-relevant equivalents of these industries include the diet-product industry, laxative industry, cosmetic surgery and procedures industry, fashion industry, advertising, and others.

Changes in the macro environment to promote public health are often achieved through regulation in the form of law and policy in the public sector or policies and practice standards in the private sector. Public health law scholar Michelle Mello describes three conditions needed to trigger regulatory action to achieve macro-environmental change to promote public health: 1) evidentiary base; 2) practical considerations; and 3) political will [31]. Mello’s framework of triggers can serve as a guide for planning a strategically coordinated public health approach to eating disorders prevention research.

### Trigger #1, evidentiary base

Mello encapsulates the first trigger with two questions: One, do economic costs favor prevention? And two, does the scientific evidence link exposure to long-term health problems? Health economists have long worked with prevention scientists to conduct cost analyses of prevention strategies to help inform policy decisions that effectively and responsibly invest public and private funds to address a range of public health problems. To date, one economic study has estimated the potential medical cost savings associated with preventing eating disorders, [5] but much more economic research is needed. Across the globe, we are living in a time of extreme fiscal austerity, and there is increasing pressure on policy makers and program planners to make sure the public health prevention efforts they champion are not only effective but also cost-effective. Building a research base to document whether and how economic costs may favor prevention of eating disorders will be an essential new line of research to advance a public health approach.

As for the evidentiary base linking exposures to health problems, this is undoubtedly the strongest area in the existing eating disorders research literature, as illustrated above with the example of the Prevention Maturation Schema (Phase 3). Using this trigger as a guide for research, cross-disciplinary teams should focus on the long-term health consequences particularly of exposures that are amenable to macro-environmental intervention, such as strengthened regulation of specific product categories (e.g., over-the-counter laxatives and diet pills and prescription stimulants, such as Adderall and Ritalin, abused for weight control), deceptive advertising to children, the cosmetic surgery and procedures industry, etc., to maximize population-wide reach and impact.

### Trigger #2, practical considerations

This trigger refers to the work of operationalizing ideas into law or policy, which requires cross-disciplinary teams of public health law scholars and eating disorders experts. One example of the product of such a cross-disciplinary collaboration is a recent detailed review of legal considerations and recommendations of viable mechanisms within the law that can be used to effectively restrict youth access to laxatives and alli® in the United States [20]. These types of teams need to work together to identify how, within a specific country’s or state/province’s regulatory system, macro-environmental forces can be targeted. The key for prevention scientists going forward is to then systematically evaluate their effect on prevention goals.

In recent years, advocates have been gaining momentum in crafting and implementing macro-environmental initiatives. In Britain, the national Advertising Standards Authority (ASA) regulates ads across all media and can compel the removal of ads that it deems misleading ( http://www.asa.org.uk/). Other policy initiatives include the Voluntary Media Code of Conduct established in 2008 by the state government in Victoria, Australia, to establish media standards related to portrayal of excessively thin models and other concerns. In Madrid, Spain, in 2006 the regional government banned employment of adult models with body mass index less than 18 kg/m^2^ to enhance occupational health and safety efforts in the industry, and similarly, the Israeli government recently banned the employment of fashion models with body mass index below 18.5 kg/m^2^[9,15,18,19]. While systematic evaluation of these policy initiative have not been reported in the literature, one new initiative in Québec, Canada, marks what may be the first time a macro-environmentally targeted intervention related to eating disorders prevention has been systematically evaluated and reported in the scientific literature [17]. The Québec Ministry of Culture, Communications, and the Status of Women led a media campaign to promote healthy body image in the province and included multiple trainings on body image and eating disorders for provincial professionals in fashion and modeling, advertising, media, retailing, manufacturing, and health care. Researchers then conducted an evaluation with the local population six months after the initiative was launched, finding that about a third of the 1003 residents surveyed recognized the campaign and had favorable views of its healthy body image promotion goals [17].

As has been done in other fields [28] and now has been done for the first time in the eating disorders prevention field with the Québec intervention [17], the critical role of prevention researchers is to evaluate the effects of policy and regulatory initiatives like the ones described above and others on eating disorders prevention. Industry-led initiatives to change practice standards and voluntary mass media codes to refrain from images and messages that stigmatize overweight children and adults [33] should be included among the interventions to be evaluated.

### Trigger #3, political will

Fostering political will requires advocacy to move eating disorders higher up on the political and social agendas for policymakers, community and business leaders, voters, and consumers. This can be done through activities such as lobbying government, community organizing, and media advocacy to shift social norms. As described above, advocates in a number of countries have been leaders in these types of activities [9,15,17,19]. The role for eating disorders prevention researchers is to work across disciplines with political scientists, media researchers and professionals, community organizers, and others to examine the effects of these initiatives. Rigorous evaluation of social norms campaigns, counter ads, media coverage, and grass-roots community organizing efforts and studies tracking shifts in the opinions and actions of the public, government officials, and industry trend setters will help to determine if strategic efforts to foster political will are successful in advancing eating disorders prevention.

### Summary

An argument has been offered above for what steps need to be taken to bring a public health approach to eating disorders prevention. First, stock was taken of what the field has achieved so far, using tools from the prevention science literature, and, second, a research plan of action is offered that plays to the unique strengths of public health, drawing on a triggers-to-action framework from public health law. Minimal participation was found from public health professionals in eating disorders prevention research, and the vast majority of prevention research to date was found to be concentrated within the disciplines of psychology and psychiatry. Extreme disciplinary concentration of the research has led to a preponderance of individually targeted prevention strategies with little research focused on environmental targets, particularly at the macro level. It was proposed that a triggers-to-action framework, which focuses on evidentiary base, practical considerations, and political will, developed in public health law can provide a strong basis for a strategic research plan for a public health approach to eating disorders prevention.

There is enormous potential for growth in the scope and diversity of eating disorders prevention research strategies, particularly those targeting the macro environment. A public health approach to eating disorders prevention research will require a strategic research plan that capitalizes on the strengths of public health by leveraging the macro environment for prevention. But first, we need public health professionals to pull up a chair.

## Competing interests

The author declares that she has no competing interests.

## Authors’ contribution

SBA was responsible for manuscript conception, data collection and analysis, and manuscript writing.

## Author information

SBA is with the Department of Society, Human Development, and Health of Harvard School of Public Health and the Division of Adolescent and Young Adult Medicine at Boston Children’s Hospital in Boston, MA. SBA is director of the Strategic Training Initiative for the Prevention of Eating Disorders at the Harvard School of Public Health and Boston Children’s Hospital ( http://www.hsph.harvard.edu/striped) and is on the editorial board of *Eating Disorders: The Journal of Treatment and Prevention*.

## Human participant protection

As a scholarly essay, this manuscript does not report original human subjects research data and therefore did not require institutional review board approval.

## Pre-publication history

The pre-publication history for this paper can be accessed here:

http://www.biomedcentral.com/1471-2458/12/854/prepub
